# Vaccine effectiveness against COVID-19 hospitalisation in adults (≥ 20 years) during Omicron-dominant circulation: I-MOVE-COVID-19 and VEBIS SARI VE networks, Europe, 2021 to 2022

**DOI:** 10.2807/1560-7917.ES.2023.28.47.2300187

**Published:** 2023-11-23

**Authors:** Angela MC Rose, Nathalie Nicolay, Virginia Sandonis Martín, Clara Mazagatos, Goranka Petrović, Joaquin Baruch, Sarah Denayer, Lucie Seyler, Lisa Domegan, Odile Launay, Ausenda Machado, Cristina Burgui, Roberta Vaikutyte, F Annabel Niessen, Isabela I Loghin, Petr Husa, Nassera Aouali, George Panagiotakopoulos, Kristin Tolksdorf, Judit Krisztina Horváth, Jennifer Howard, Francisco Pozo, Virtudes Gallardo, Diana Nonković, Aušra Džiugytė, Nathalie Bossuyt, Thomas Demuyser, Róisín Duffy, Liem binh Luong Nguyen, Irina Kislaya, Iván Martínez-Baz, Giedre Gefenaite, Mirjam J Knol, Corneliu Popescu, Lenka Součková, Marc Simon, Stella Michelaki, Janine Reiche, Annamária Ferenczi, Concepción Delgado-Sanz, Zvjezdana Lovrić Makarić, John Paul Cauchi, Cyril Barbezange, Els Van Nedervelde, Joan O’Donnell, Christine Durier, Raquel Guiomar, Jesús Castilla, Indrė Jonikaite, Patricia CJL Bruijning-Verhagen, Mihaela Lazar, Regina Demlová, Gil Wirtz, Marina Amerali, Ralf Dürrwald, Mihály Pál Kunstár, Esther Kissling, Sabrina Bacci, Marta Valenciano, I-MOVE-COVID-19 hospital study team, VEBIS hospital study team, Members of the I-MOVE-COVID-19 and VEBIS hospital study teams (in addition to authors above), Svjetlana Karabuva, Suzana Mladinov, Joško Markić, Petra Tomaš Petrić, Irena Tabain, Maja Ilić, Ivan Mlinarić, Petra Smoljo, Iva Pem Novosel, Tanya Melillo, Maria-Louise Borg, Benédicte Lissoir, Xavier Holemans, Marc Hainaut, Nicolas Dauby, Benedicte Delaere, Marc Bourgeois, Evelyn Petit, Marijke Reynders, Door Jouck, Koen Magerman, Marieke Bleyen, Melissa Vermeulen, Sébastien Fierens, François Dufrasne, Siel Daelemans, Ala’a Al Kerwi, Francoise Berthet, Guy Fagherazzi, Myriam Alexandre, Charlene Bennett, Jim Christle, Jeff Connell, Peter Doran, Laura Feeney, Binita Maharjan, Sinead McDermott, Rosa McNamara, Nadra Nurdin, Zineb Lesieur, Salif Mamadou Cissé, Anne-Sophie L'Honneur, Xavier Duval, Yolande Costa, Fidouh Nadhira, Florence Galtier, Laura Crantelle, Vincent Foulongne, Phillipe Vanhems, Sélilah Amour, Bruno Lina, Fabrice Lainé, Laetitia Gallais, Gisèle Lagathu, Anna Maisa, Sibylle Bernard-Stoecklin, Yacine Saidi, Christine Durier, Rebecca Bauer, Ana Paula Rodrigues, Adriana Silva, Verónica Gomez, Margarida Tavares, Débora Pereira, Maria José Manata, Heidi Gruner, André Almeida, Paula Pinto, Cristina Bárbara, Ana Miqueleiz, Ana Navascués, Camino Trobajo-Sanmartín, Carmen Ezpeleta, Nerea Egüés, Manuel García Cenoz, Eva Ardanaz, Marcela Guevara, Conchi Moreno-Iribas, Itziar Casado, Auksė Mickiene, Monika Kuliese, Hana Orlíková, Carmen Mihaela Dorobat, Carmen Manciuc, Simin Aysel Florescu, Alexandru Marin, Sorin Dinu, Catalina Pascu, Alina Ivanciuc, Iulia Bistriceanu, Mihaela Oprea, Maria Elena Mihai, Silke Buda, Ute Preuss, Marianne Wedde, Beatrix Oroszi, Katalin Krisztalovics, Gergő Túri, Katalin Kristóf, Csaba Varga, Ruoran Li, Alain Moren, Anthony Nardone

**Affiliations:** 1 Epiconcept, Paris, France; 2 European Centre for Disease Prevention and Control, Stockholm, Sweden; 3 National Centre for Microbiology, Institute of Health Carlos III, Madrid, Spain; 4 Consortium for Biomedical Research in Epidemiology and Public Health (CIBERESP), Madrid, Spain; 5 National Centre for Epidemiology, Institute of Health Carlos III, Madrid, Spain; 6 Croatian Institute of Public Health, Zagreb, Croatia; 7 IDCU within Health promotion and disease prevention Directorate, G’mangia, Malta; 8 Sciensano, Brussels, Belgium; 9 Universitair Ziekenhuis Brussel, Brussels, Belgium; 10 Health Service Executive–Health Protection Surveillance Centre, Dublin, Ireland; 11 Faculty of Medicine, University of Paris City, Paris, France; 12 AP–HP, Hôpital Cochin, Paris, France; 13 Inserm, CIC Cochin-Pasteur, Paris, France; 14 National Institute of Health Dr Ricardo Jorge, Lisbon, Portugal; 15 Instituto de Salud Pública de Navarra-IdiSNA, Pamplona, Spain; 16 Lithuanian University of Health Sciences, Kaunas, Lithuania; 17 Centre for Infectious Disease Control, National Institute for Public Health and the Environment, Bilthoven, the Netherlands; 18 Grigore T. Popa University of Medicine and Pharmacy, Iasi, Romania; 19 St. Parascheva Clinical Hospital of Infectious Diseases, Iasi, Romania; 20 University Hospital Brno, Brno, Czechia; 21 Faculty of Medicine, Masaryk University, Brno, Czechia; 22 Luxembourg Institute of Health, Luxembourg; 23 National Public Health Organisation (EODY), Athens, Greece; 24 Robert Koch Institute, Berlin, Germany; 25 National Laboratory for Health Security, Epidemiology and Surveillance Centre, Semmelweis University, Budapest, Hungary; 26 Dirección General de Salud Pública y Ordenación Farmacéutica, Junta de Andalucía, Spain; 27 Teaching Public Health Institute of Split-Dalmatia County, Split, Croatia; 28 Faculty of Medicine, Lund University, Lund, Sweden; 29 Carol Davila University of Medicine and Pharmacy, Bucharest, Romania; 30 Dr Victor Babes Clinical Hospital of Infectious and Tropical Diseases, Bucharest, Romania; 31 Centre Hospitalier de Luxembourg, Luxembourg; 32 Inserm, US19, Villejuif, Paris, France; 33 Julius Center for Health Sciences and Primary Care, University Medical Center Utrecht, Utrecht, the Netherlands; 34 “Cantacuzino” National Military Medical Institute for Research-Development, Bucharest, Romania; 35 The members of these groups are listed under Collaborators

**Keywords:** COVID-19, SARS-CoV-2, hospital, omicron, vaccine effectiveness, Europe

## Abstract

**Introduction:**

The I-MOVE-COVID-19 and VEBIS hospital networks have been measuring COVID-19 vaccine effectiveness (VE) in participating European countries since early 2021.

**Aim:**

We aimed to measure VE against PCR-confirmed SARS-CoV-2 in patients ≥ 20 years hospitalised with severe acute respiratory infection (SARI) from December 2021 to July 2022 (Omicron-dominant period).

**Methods:**

In both networks, 46 hospitals (13 countries) follow a similar test-negative case–control protocol. We defined complete primary series vaccination (PSV) and first booster dose vaccination as last dose of either vaccine received ≥ 14 days before symptom onset (stratifying first booster into received < 150 and ≥ 150 days after last PSV dose). We measured VE overall, by vaccine category/product, age group and time since first mRNA booster dose, adjusting by site as a fixed effect, and by swab date, age, sex, and presence/absence of at least one commonly collected chronic condition.

**Results:**

We included 2,779 cases and 2,362 controls. The VE of all vaccine products combined against hospitalisation for laboratory-confirmed SARS-CoV-2 was 43% (95% CI: 29–54) for complete PSV (with last dose received ≥ 150 days before onset), while it was 59% (95% CI: 51–66) after addition of one booster dose. The VE was 85% (95% CI: 78–89), 70% (95% CI: 61–77) and 36% (95% CI: 17–51) for those with onset 14–59 days, 60–119 days and 120–179 days after booster vaccination, respectively.

**Conclusions:**

Our results suggest that, during the Omicron period, observed VE against SARI hospitalisation improved with first mRNA booster dose, particularly for those having symptom onset < 120 days after first booster dose.

**Key public health message**

**What did you want to address in this study?**

In order to understand how well the COVID-19 vaccine is performing in Europe against hospitalisation during the period when the SARS-CoV-2 Omicron variant was circulating, we investigated vaccine effectiveness using data from a multi-country study of complete and booster-dose COVID-19 vaccination among adults aged 20 years and over.

**What have we learnt from this study?**

Between December 2021 and July 2022, vaccine effectiveness against hospitalisation with laboratory-confirmed SARS-CoV-2 was 43% for complete vaccination. With addition of an mRNA booster dose, effectiveness was 59% overall. It was higher when onset of illness was close to the date of the last vaccination, at 85% when last booster dose was 14–59 days before onset, at 70% for 60–119 days, and falling below 40% for 120–179 days.

**What are the implications of your findings for public health?**

In European hospital settings in 2022, during the Omicron period, COVID-19 mRNA booster vaccine provided an improved benefit for preventing hospitalisation, particularly if disease onset was within 4 months of receiving the booster dose.

## Introduction

Safe and effective COVID-19 vaccines were a powerful tool for the control of the COVID-19 pandemic, along with non-pharmaceutical interventions to reduce transmission of severe acute respiratory syndrome coronavirus 2 (SARS-CoV-2). By the end of 2021 in the European Union (EU)/European Economic Area (EEA), one subunit and four spike protein-based COVID-19 vaccines had a conditional marketing authorisation for use [1]: the mRNA vaccines Comirnaty (BNT162b2; Pfizer-BioNTech) and Spikevax (mRNA-1273; Moderna), the adenoviral vector vaccines Vaxzevria (AZD1222; AstraZeneca) and Jcovden (Ad26.COV 2.5; Johnson & Johnson), and Nuvaxovid (NVX-CoV2373; Novavax), a subunit vaccine. All vaccines deployed in the early phase of the EU vaccination campaign were reported to be highly efficacious in randomised clinical trials [2-4]. The effectiveness of complete primary series vaccination (PSV) in real-life, post-authorisation studies was high [5-8]. Hospitalisation rates in patients ≥ 80 years in the EU/EEA declined by between 46% and 78% (depending on the level of the country’s vaccine uptake) after vaccine deployment [9], which also averted > 450,000 deaths in those aged 60 years and over [10].

Since the beginning of its circulation, SARS-CoV-2 has been evolving, with new variants emerging. The initial real-life effectiveness studies were performed in 2021 during the circulation of the Alpha and Delta variants of concern. The emergence of the Omicron variant late in 2021 emphasised the indication of a first booster dose [11,12]. The rationale behind this was from evidence of waning of neutralising vaccine antibodies, which could cause a loss of protection against SARS-CoV-2 [13,14], shown by a decline in vaccine effectiveness (VE) over time against infection and hospitalisation [15,16]. Although the objective of PSV was to prevent or reduce the occurrence of all COVID-19 outcomes [5,17,18], the administration of a booster dose aimed to restore the protection against severe outcomes conferred by primary vaccination at time of Delta predominance [19]. By December 2021, all EU/EEA countries recommended a booster dose after complete PSV of authorised vaccines for adults [20], with many recommending a delay of 150–180 days between last PSV dose and booster [21]. Priority groups for the administration of a booster dose included individuals at risk of severe COVID-19 outcomes based on presence of underlying medical condition or age with an age-based staggered approach from older to younger age groups. By 30 June 2022, the vaccination coverage rates for first booster dose had reached 83% in ≥ 80-year-olds compared with 39% in 18–24-year-olds in the EU/EEA countries [22].

Monitoring of VE against specific sub-lineages is vital to inform public health guidance for COVID-19 vaccination. In Europe, multicentre hospital-based studies have been implemented to monitor VE over time [23]. We aimed to estimate COVID-19 VE using pooled data from the Vaccine Effectiveness, Burden and Impact (VEBIS) hospital severe acute respiratory infection (SARI) VE network [24] and the I-MOVE-COVID-19 network [25]. At the end of 2021, the SARS-CoV-2 Omicron variant began to dominate in countries participating in these networks. 

We aimed to estimate VE of complete PSV and first booster dose against hospitalisation with PCR-confirmed SARS-CoV-2 among patients aged ≥ 20 years with SARI swabbed between 13 December 2021 and 31 July 2022 (Omicron-dominant period).

## Methods

### Setting

**Figure 1:**
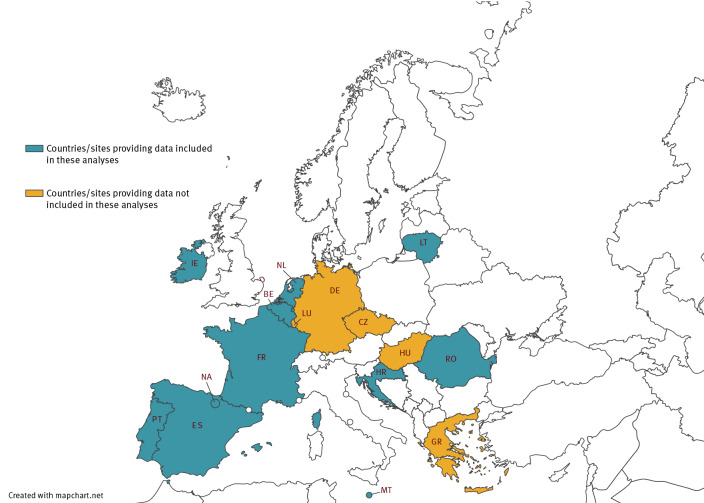
Countries and study sites participating in I-MOVE-COVID-19 and VEBIS hospital vaccine effectiveness studies, by provision of data for this analysis, Europe, 2021–2022

I-MOVE: Influenza – Monitoring Vaccine Effectiveness in Europe; SARS-CoV-2: severe acute respiratory syndrome coronavirus 2; VEBIS: Vaccine Effectiveness, Burden and Impact Studies.

The two hospital networks (I-MOVE-COVID-19 and ECDC VEBIS) include 48 hospitals across 16 sites in 15 countries (with two sites in Spain; Figure 1).

Sixteen participating sites*:* Belgium (BE), Croatia (HR), Czechia (CZ), France (FR), Germany (DE), Greece (GR), Hungary (HU), Ireland (IE), Lithuania (LT), Luxembourg (LU), Malta (MT), the Netherlands (NL), Portugal (PT), Romania (RO), Spain 11 regions (ES), Spain Navarra region (NA). Eleven included in this analysis: BE, ES, FR, HR, IE, LT, MT, NA, NL, PT, RO.

### Study design and case definitions

Hospitals participating in both networks share similar generic protocols [24,25] and use the test-negative case–control design [26]. Demographic and clinical data and COVID-19 vaccination information were collected via questionnaire, electronic medical records or patient interview, depending on the site. Study sites collected information on all, or a systematic sample of, hospitalised SARI patients (for details see [27]).

We defined a SARI patient as an individual hospitalised for ≥ 24 h with at least one symptom (fever, cough, shortness of breath, sudden onset anosmia, ageusia or dysgeusia) using the ECDC definition for a possible COVID-19 case [28]. Cases were SARI patients PCR-positive for SARS-CoV-2 within 48 h of admission or in the 14 days before hospital admission. Controls were SARI patients PCR-negative at or within 48 h of admission. Controls with known SARS-CoV-2 infection confirmed by PCR within 14 days of onset were re-coded as cases. We used time of swab as a proxy for start of Omicron and Omicron sub-lineage dominance, defined as the first day of the country-specific week in which ≥ 80% of all samples available in GISAID [29,30] were identified as B.1.1.529 (or Omicron BA.1, BA.2 and BA.4/5), up to the end of the study period (31 July 2022).

### Inclusion and exclusion criteria

We excluded patients with missing/erroneous key variables (age, sex, key dates, vaccination information). We also excluded (i) patients not in each country’s age-specific booster dose target group (BDTG) at the time of swab, (ii) those with non-homologous PSV (for two-dose schedules; counting Comirnaty and Spikevax as ‘homologous’ as both are mRNA vaccines) and (iii) those with mRNA booster vaccination who had received this dose < 150 days after their last PSV dose. We did not include in the analysis data from sites with fewer than five cases/controls or fewer than 20 total cases and controls.

### Definitions of vaccination status 

We defined complete PSV as two doses of a two-dose vaccine schedule (or three for immunocompromised individuals) or one dose of Jcovden, and booster vaccination as complete PSV plus first mRNA booster dose. Vaccination was considered valid only if vaccines were received ≥ 14 days before symptom onset and with recommended delays between doses (those vaccinated < 14 days before symptom onset, and those with non-recommended delays between doses were excluded).

### Statistical analysis

We compared the odds of vaccination between cases and controls using logistic regression, calculating VE as 1 minus the odds ratio (OR) of vaccination among cases and controls (expressed as a percentage). We included study site (as a fixed effect) and date of swab (modelled as a spline or categorical variable, with the best functional form designated by the Akaike information criterion) in all VE analyses. We further adjusted the OR by age (modelled as a spline, a linear term, or as a categorical age group variable, with the best functional form designated by the Akaike information criterion), by sex and by presence of at least one of four commonly collected chronic conditions (asthma, diabetes, heart disease, lung disease) or absence of all four.

We estimated absolute VE during the Omicron period for complete PSV only and for complete PSV plus first mRNA booster dose, vs no COVID-19 vaccination. For complete PSV analyses, we included vaccinated patients in two main groups: those not yet eligible for booster, having received their last PSV dose < 150 days prior (Analyses 1–2), and those eligible for booster, with last PSV dose ≥ 150 days before onset of symptoms (Analyses 3–5). The 150-day period was selected as, in general, the recommendation in participating countries was for booster to be administered 5 months after last PSV dose.

In Analysis 1, we estimated VE for all products combined in those who had received complete PSV only with symptom onset < 150 days of the last PSV dose.

In Analysis 2, we estimated booster dose VE for those with at least 150 days between last PSV dose and booster dose. For comparison, we estimated VE in those who had not received a booster dose, but who had received their last PSV dose ≥ 150 days before onset. We estimated VE overall, by age group and by PSV product.

In Analysis 3, we estimated VE by time since vaccination for those receiving a first mRNA booster dose for all ages combined, using the intervals 14–59, 60–119, 120–179 and ≥ 120 days from receipt of the booster (overall and by PSV product). In Analysis 4 we also estimated VE by time since vaccination, for the same periods as Analysis 3, but stratified into three age groups (20–59, 60–79 and ≥ 80 years), for all PSV products combined. In both of these analyses, we only included vaccinated SARI patients for whom the delay between last PSV dose and booster dose was ≥ 150 days.

In supplementary analyses, we estimated VE by time since last PSV dose for each of three periods (14–59, 60–119 and 120–149 days). Further, we measured VE for the periods of dominance of each of the Omicron variant sub-lineages BA.1, BA.2 and BA.4/5, overall and by vaccine category/product, age group, presence of at least one chronic condition and time since booster dose vaccination (< 60 days, 60–119 days, ≥ 120 days).

### Sensitivity analyses

We performed sensitivity analyses (i) excluding all SARI patients with known prior infection > 14 days before onset and (ii) including those with severe outcomes only (admission to an intensive care unit (ICU) or in-hospital death). Where the number of cases or controls per parameter was < 10, we conducted sensitivity analysis using Firth’s method of penalised logistic regression (PLR) to assess small sample bias [31,32]. We do not show estimates with a difference between the PLR and original VE estimate > 10 percentage points.

## Results

### Descriptive analysis

**Figure 2:**
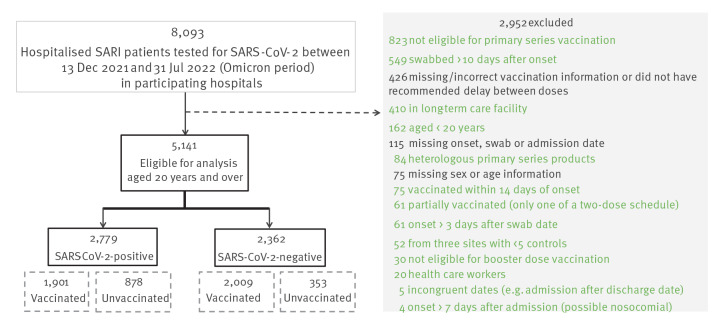
Exclusions, I-MOVE-COVID-19 and VEBIS hospital vaccine effectiveness studies, Europe, 2021–2022 (n = 8,093)

I-MOVE: Influenza – Monitoring Vaccine Effectiveness in Europe; SARS-CoV-2: severe acute respiratory syndrome coronavirus 2; VEBIS: Vaccine Effectiveness, Burden and Impact Studies.

After applying exclusion criteria (Figure 2), we included 2,779 cases and 2,362 controls aged ≥ 20 years from 40 of 48 European hospitals in 11 of 16 participating study sites providing sufficient data (at least five cases and controls) for the study period (Figure 1).

Included data are from 40 hospitals in 11 participating sites.

The highest proportion of cases by age group were those ≥ 80 years (1,212; 44% vs 872 controls; 37%); 1,187 (43%) cases and 1,068 (45%) controls were female; 1,884 (68%) cases and 1,773 (75%) controls had at least one of the four listed chronic conditions (Table 1). For this analysis, 1,570 SARI patients (31%) were swabbed during the Omicron BA.1 sub-lineage period (13 December 2021–20 February 2022), 1,309 (25%) during the BA.2 period (7 March–29 May 2022) and 606 (12%) in the BA.4/5 period (10 June–31 July 2022). The remaining 1,656 (32%) were swabbed during a period undefined by these sub-lineages (Table 1).

**Table 1:** Characteristics of cases and controls, I-MOVE-COVID-19 and VEBIS vaccine effectiveness hospital networks, Europe, Omicron period December 2021–July 2022 (n = 5,141)

Patient characteristic	SARS-CoV-2 cases (n = 2,779)		Test negative controls (n = 2,362)	
	Number	%	Number	%
Median age (years)	77		75	
Age groups (years)				
20–59	420	15.1	439	18.6
60–79	1,147	41.3	1,051	44.5
≥ 80	1,212	43.6	872	36.9
Sex				
Male	1,592	57.3	1,294	54.8
Female	1,187	42.7	1,068	45.2
At least one chronic condition^a^				
No	895	32.2	589	24.9
Yes	1,884	67.8	1,773	75.1
Omicron sub-lineage periods^b^				
Swab date 13 December 2021–20 February 2022 (BA.1)	1,067	38.4	503	21.3
Swab date 7 March–29 May 2022 (BA.2)	554	19.9	755	32.0
Swab date 10 June–31 July 2022 (BA.4/5)	399	14.4	207	8.8
Sub-lineage period undefined^c^	759	27.3	897	38.0
COVID-19 vaccination status				
Unvaccinated	878	31.6	353	14.9
Complete PSV only	502	18.1	395	16.7
Complete PSV + first booster only	1,389	50.0	1,598	67.7
Complete PSV + two boosters	10	0.4	16	0.7
Vaccine product among vaccinated: first dose				
Comirnaty	1,480	77.9	1,508	75.1
Vaxzevria	200	10.5	256	12.7
Spikevax	158	8.3	185	9.2
Janssen	62	3.3	53	2.6
Other/unknown	1	0.1	7	0.3
Vaccine product among vaccinated: second dose				
Comirnaty	1,482	80.6	1,497	76.5
Vaxzevria	200	10.9	256	13.1
Spikevax	156	8.5	196	10.0
Other/unknown	1	0.1	7	0.4
Vaccine product among vaccinated: first booster dose				
Comirnaty	1,096	78.3	1,155	71.6
Spikevax	299	21.4	446	27.6
Other/unknown	4	0.2	13	0.8
Vaccine product among vaccinated: second booster dose				
Comirnaty	8	80.0	12	75.0
Spikevax	2	20.0	3	18.8
Other/unknown	0	0.0	1	6.3
Study site and country^d^				
Belgium	161	5.8	223	9.4
Croatia	515	18.5	167	7.1
Czechia	NI			
France	112	4.0	91	3.9
Germany	NI			
Greece	NI			
Hungary	NI			
Ireland	96	3.5	109	4.6
Lithuania	23	0.8	49	2.1
Luxembourg	NI			
Malta	185	6.7	261	11.0
the Netherlands	104	3.7	166	7.0
Portugal	78	2.8	58	2.5
Romania	83	3.0	5	0.2
Spain (Navarra)	111	4.0	84	3.6
Spain (11 regions)	1,311	47.2	1,149	48.6
Days from vaccination (any vaccine product)	Median	IQR	Median	IQR
Last PSV dose to onset (patients with PSV only; including those with last dose < 150 days from symptom onset)	240	183–303	244	190–310
Last PSV dose to onset (patients with PSV only; excluding those with last dose < 150 days from onset)	254	208–314	253	209–319
First booster dose to symptom onset	159	98–208	127	81–176
Last PSV dose to first booster dose^e^	209	189–231	206	188–229

ICU: intensive care unit; I-MOVE: Influenza – Monitoring Vaccine Effectiveness in Europe; IQR: inter-quartile range; NI: no data included; PSV: primary series vaccination; SARS-CoV-2: severe acute respiratory syndrome coronavirus 2; VEBIS: Vaccine Effectiveness, Burden and Impact Studies.

**^a^** At least one of four commonly collected chronic conditions: asthma, diabetes, heart disease, lung disease.

**^b^** Omicron sub-lineage period defined using proxy dates in each study country to mark the week in which the proportion of samples sequenced and reported to GISAID reached ≥ 80% for the start of the period, and the end of the week before the one in which < 80% of samples sequenced were from a particular sub-lineage (BA.1, BA.2 and either BA.4 or BA.5).

^c^ Sub-lineage period undefined means all those SARI patients with onset dates that did not fall within any of the defined sub-lineage periods above.

^d^ Three sites submitting data during the Omicron period did not have a sufficient number of eligible SARI patients for inclusion in these analyses, and two sites did not submit data for this period (this table gives final numbers by site, after exclusions).

^e^ Note that patients with booster dose < 150 days from last PSV dose were excluded from analyses.

At the time of swab, 878 cases (32%) and 353 controls (15%) were unvaccinated. Half of the cases (n = 1,389) and 1,598 controls (68%) had received both complete PSV and first booster doses; 10 cases (0.4%) and 16 controls (0.7%) had a second booster dose (Table 1). Seventy-eight per cent of cases and 75% of controls were vaccinated with Comirnaty for their first dose; 81% and 77% for the second dose, respectively.

**Figure 3:**
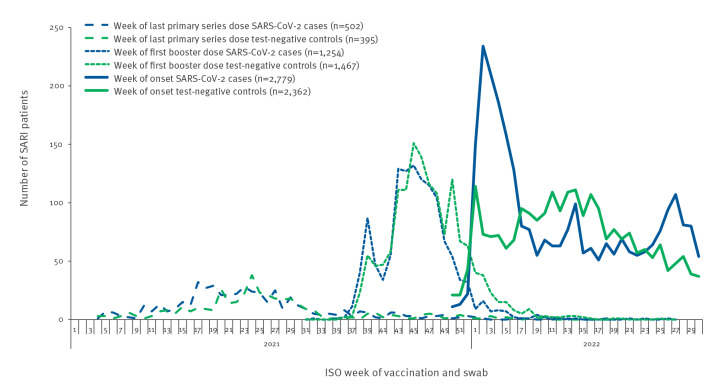
Number of SARI patients by case status and week of COVID-19 vaccination or swab (complete primary series and booster doses), by vaccine product, I-MOVE-COVID-19 and VEBIS hospital vaccine effectiveness studies, Europe, 2021–2022 (n = 5,141)

I-MOVE: Influenza – Monitoring Vaccine Effectiveness in Europe; SARI: severe acute respiratory infection; SARS-CoV-2: severe acute respiratory syndrome coronavirus 2; VEBIS: Vaccine Effectiveness, Burden and Impact Studies.

SARI patients received their last PSV dose between week 4, 2021 and week 18, 2022 (Figure 3). The median time from last PSV dose to onset of symptoms for patients with complete PSV only was 240 days (interquartile range (IQR): 183–303) in cases and 244 days (IQR: 190–310) in controls (Table 1). Excluding those with last PSV dose < 150 days of onset, these medians were 254 days (IQR: 208–314) and 253 days (IQR: 209–319), respectively. SARI patients received their first booster doses between week 21, 2021 and week 26, 2022. The median time from first booster dose to onset was 160 days (IQR: 101–211) for cases and 132 days (IQR: 86–181) for controls. The median time between last PSV dose and first booster dose (in those with booster dose ≥ 150 days after last complete PSV dose) was 209 days (IQR: 189–231) for cases, 206 days (IQR: 188–229) for controls.

### Analysis 1: Vaccine effectiveness for primary series vaccination in individuals not yet eligible for a booster dose 

We estimated VE among individuals who had received their last PSV dose <150 days prior to symptom onset. Among this group, VE against hospitalisation with COVID-19 for complete PSV was 51% (95% confidence interval (CI): 21–69) for all PSV products combined (Table 2). For age-stratified and time-since-vaccination results for this group, see Supplementary Table S1.

**Table 2:** Effectiveness of COVID-19 complete primary series vaccination and booster vaccination against hospitalisation among adults (≥ 20 years) by age group and vaccine product, I-MOVE-COVID-19 and VEBIS hospital vaccine effectiveness studies, Europe, 13 December 2021–31 July 2022 (n = 5,115^a^)

PSV vaccine product	Vaccinated/unvaccinated cases; Vaccinated/unvaccinated controls			VE^b^ (95% CI)		
Analysis 1: VE < 150 days from receipt of last PSV dose to symptom onset in those not eligible for booster dose						
Any PSV vaccine product (eight sites^c^; n = 1,245^d^)						
All 14–149 days	67/791; 48/339			51 (21 to 69)		
Analysis 2: VE in those eligible for first booster dose. Vaccination ≥ 150 days before symptom onset for patients with PSV only and ≥ 150 days between last PSV and booster dose (for those with PSV + mRNA booster)						
	Complete PSV only		Complete PSV plus mRNA booster	Complete PSV only		Complete PSV plus mRNA booster
**Any PSV product**	**11 sites^e^; n = 2,007^f^**		**10 sites^g^; n = 3,876^h^**			
All ≥ 20	430/878; 346/353		1,249/811; 1,467/349	43 (29 to 54)		59 (51 to 66)
**Age group**						
20–59	103/179; 137/97		83/165; 161/94	65 (46 to 77)		66 (47 to 78)
60–79	193/382; 143/155		460/349; 643/154	38 (13 to 56)		58 (45 to 68)
≥ 80	134/317; 66/101		706/297; 663/101	29 (−7 to 53)		58 (42 to 69)
**Chronic condition^i^**						
No	146/374; 121/119		292/346; 279/116	60 (43 to 72)		64 (48 to 75)
Yes	284/504; 225/234		957/465; 1,188/233	32 (11 to 48)		56 (45 to 64)
**Comirnaty PSV**	**10 sites^g^; n = 1,670**		**10 sites^g^; n = 3,401**			
All ≥ 20	282/802; 240/346		1,079/811; 1,162/349	49 (34 to 60)		54 (45 to 62)
**Age group**						
20–59	79/165; 89/93		56/165; 101/94	58 (33 to 73)		59 (34 to 74)
60–79	104/342; 98/153		371/349; 461/154	53 (29 to 68)		52 (36 to 64)
≥ 80	99/295; 53/100		652/297; 600/101	27 (−16 to 54)		54 (37 to 66)
**Chronic condition^i^**						
No	90/341; 82/114		248/346; 205/116	64 (45 to 76)		60 (41 to 72)
Yes	192/461; 158/232		831/465; 957/233	34 (11 to 52)		52 (39 to 61)
**Spikevax PSV**	**Eight sites^c^; n = 1,275**		**10 sites^g^; n = 1,382**			
All ≥ 20	42/855; 34/344		92/811; 130/349	50 (14 to 71)		60 (44 to 71)
**Age group**						
20–59	7/174; 17/91		12/165; 31/94	76 (32 to 92)		69 (32 to 86)
60–79	25/370; 12/154		38/349; 52/154	23 (−77 to 66)		54 (21 to 73)
≥ 80	10/311; 5/99		42/297; 47/101	NC		58 (26 to 76)
**Chronic condition^i^**						
No	14/360; 10/116		17/346; 29/116	64 (5 to 86)		75 (49 to 88)
Yes	28/495; 24/228		75/465; 101/233	39 (−17 to 68)		50 (27 to 66)
**Vaxzevria PSV**	**Nine sites^j^; n = 1,263**		**Nine sites^j^; n = 1,355**			
All ≥ 20	72/802; 43/346		60/802; 147/346	30 (−10 to 56)		69 (54 to 79)
**Age group**						
20–59	5/165; 12/93		11/165; 23/93	NC		71 (25 to 89)
60–79	51/342; 25/153		39/342; 110/153	−5 (−95 to 44)		68 (47 to 81)
≥ 80	16/295; 6/100		10/295; 14/100	42 (-68 to 80)		51 (−54 to 84)
**Chronic condition^i^**						
No	26/341; 17/114		21/341; 40/114	58 (13 to 80)		71 (43 to 86)
Yes	46/461; 26/232		39/461; 107/232	15 (−52 to 53)		69 (51 to 80)
Analysis 3: VE by time (number of days) since receipt of first mRNA booster dose (14–59, 60–119, 120–179, ≥ 180 days from receipt of booster to symptom onset) by PSV product						
**All PSV products combined (10 sites^g^; n = 3,876^h^)**						
All ≥ 14 days from first mRNA booster dose	1,249/811; 1,467/349			59 (51 to 66)		
**Days from first mRNA booster dose to onset (among vaccinated cases and controls)**						
*14–59*	115/811; 212/349			85 (78 to 89)		
*60–119*	304/811; 464/349			70 (61 to 77)		
*120–179*	328/811; 444/349			36 (17 to 51)		
*≥ 180*	502/811; 347/349			−3 (−37 to 23)		
**Comirnaty PSV (10 sites^g^; n = 3,401)**						
All ≥ 14 days from first mRNA booster dose	1,079/811; 1,162/349			54 (45 to 62)		
**Days from first mRNA booster dose to onset**						
14–59	89/811; 164/349			86 (79 to 90)		
60–119	260/811; 340/349			66 (56 to 74)		
120–179	280/811; 358/349			34 (13 to 50)		
≥ 180	450/811; 300/349			−8 (−45 to 20)		
**Spikevax PSV (10 sites^g^; n = 1,382)**						
All ≥ 14 days from first mRNA booster dose	92/811; 130/349			60 (44 to 71)		
**Days from first mRNA booster dose to onset**						
14–59	10/811; 22/349			85 (65 to 93)		
60–119	20/811; 40/349			79 (61 to 89)		
120–179	29/811; 37/349			27 (−28 to 58)		
≥ 180	33/811; 31/349			27 (−29 to 59)		
**Vaxzevria PSV (nine sites;^j^ n = 1,355)**						
All ≥ 14 days from first mRNA booster dose	60/802; 147/346			69 (54 to 79)		
**Days from first mRNA booster dose to onset**						
14–59	14/802; 20/346			75 (45 to 89)		
60–119	18/802; 77/346			78 (60 to 88)		
120–179	16/802; 36/346			43 (−11 to 71)		
≥ 180	12/802; 14/346			27 (−74 to 70)		
Analysis 4: VE by time (number of days) since receipt of mRNA booster dose (14–59, 60–119, 120–179, ≥ 180 days from receipt of booster dose to symptom onset) for all PSV products combined, by age group (20–59, 60–79, ≥ 80 years)						
**Aged 20–59 years (10 sites^g^; n = 503)**						
All ≥ 14 days from first mRNA booster dose		83/165; 161/94			66 (47 to 78)	
**Days from first mRNA booster dose to onset (among vaccinated cases and controls)**						
14–59		23/165; 47/94			81 (62 to 91)	
60–119		27/165; 69/94			59 (25 to 78)	
120–179		17/165; 30/94			38 (−33 to 71)	
≥ 180		16/165; 15/94			−35 (−257 to 49)	
**Aged 60–79 years (10 sites^g^; n = 1,606)**						
All ≥ 14 days from first mRNA booster dose		460/349; 643/154			58 (45 to 68)	
**Days from first mRNA booster dose to onset (among vaccinated cases and controls)**						
14–59		56/349; 105/154			85 (75 to 91)	
60–119		113/349; 209/154			70 (55 to 80)	
120–179		135/349; 200/154			28 (−7 to 52)	
≥ 180		156/349; 129/154			2 (−59 to 40)	
**Aged ≥ 80 years (10 sites^g^; n = 1,767)**						
All ≥ 14 days from first mRNA booster dose		706/297; 663/101			58 (42 to 69)	
**Days from first mRNA booster dose to onset (among vaccinated cases and controls)**						
14–59		36/297; 60/101			86 (73 to 93)	
60–119		164/297; 186/101			76 (63 to 85)	
120–179		176/297; 214/101			38 (5 to 59)	
≥ 180		330/297; 203/101			−4 (−59 to 32)	

CI: confidence interval; ICU: intensive care unit; I-MOVE: Influenza – Monitoring Vaccine Effectiveness in Europe; PSTG: primary series target group; PSV: primary series vaccination; VE: vaccine effectiveness; VEBIS: Vaccine Effectiveness, Burden and Impact Studies.

^a^ n = 5,115 after dropping 26 with a second booster (sample size too small to analyse second booster).

^b^ Odds ratio adjusted by country, time (restricted cubic spline of swab date or swab month as categorical variable, depending on model), age (restricted cubic spline or age as linear variable, depending on model), sex, presence/absence of chronic condition (asthma, diabetes, heart disease, lung disease).

^c^ Eight sites: Belgium, Croatia, France, Ireland, Malta, the Netherlands, Portugal and Spain.

^d^ n = 1,245 after dropping 2,987 records of patients who received a first mRNA booster dose, 776 with last primary series vaccination dose ≥ 150 days from onset and 107 from three sites with fewer than five controls or fewer than 20 SARI patients.

^e^ Eleven sites: Belgium, Croatia, France, Ireland, Lithuania, Malta, Navarra, the Netherlands, Portugal, Romania and Spain.

^f^ n = 2,007 after dropping 2,987 records of patients who received a first mRNA booster dose, and 266 with last primary series vaccination dose < 150 days from onset.

^g^ Ten sites: Belgium, Croatia, France, Ireland, Lithuania, Malta, Navarra, the Netherlands, Portugal and Spain.

^h^ n = 3,876 after dropping 897 records from patients who had received primary series vaccination only (no booster), 262 who had received their booster dose < 150 days before symptom onset, and 76 from one site with fewer than five controls.

^I^ In analyses stratified by chronic condition, the adjustment for presence/absence of chronic condition was removed.

^j^ Nine sites: Belgium, Croatia, France, Ireland, Lithuania, Malta, the Netherlands, Portugal and Spain.

### Analysis 2: Vaccine effectiveness of primary series vaccination with or without first mRNA booster 

We estimated VE among individuals eligible for booster and with ≥ 150 days between last PSV and symptom onset (for PSV alone) or booster dose (for PSV plus booster). For all PSV products combined, VE in those who received complete PSV alone was 43% (95% CI: 29–54). In those receiving complete PSV and an mRNA booster dose, VE was 59% (95% CI: 51–66). With Comirnaty as PSV, the VE for any mRNA booster was 54% (95% CI: 45–62); it was 60% (95% CI: 41–72) with Spikevax as PSV and 69% (95% CI: 54–79) with Vaxzevria as PSV. For those aged 20–59, 60–79 and ≥ 80 years, mRNA booster dose VE for all PSV products combined was 66% (95% CI: 47–78), 58% (95% CI: 45–68) and 56% (95% CI: 45–64), respectively. The VE for all PSV products combined in those without any of the four commonly collected chronic conditions was 60% (95% CI: 43–72) without and 64% (95% CI: 48–75) with mRNA booster dose. For those with at least one of these chronic conditions, these VE were 32% (95% CI: 11–48) and 56% (95% CI: 45–64), respectively (Table 2).

### Analysis 3: Vaccine effectiveness by time since mRNA booster dose 

We estimated VE among those receiving a first booster dose for the intervals 14–59, 60–119, 120–179 and ≥ 180 days between first booster dose and symptom onset. For all PSV products combined, VE was 85% (95% CI: 78–89) in those receiving the first mRNA booster dose 14–59 days before symptom onset. For those with mRNA booster 120–179 days before onset, VE was 36% (95% CI: 17–51). For Comirnaty and Spikevax PSV, followed by first mRNA booster dose 14–59 days before symptom onset, VE point estimates were ≥ 85%; for Vaxzevria PSV plus mRNA booster 14–59 days before onset, VE was 75% (95% CI: 45–89). For those receiving Vaxzevria, Comirnaty and Spikevax as PSV followed by a first mRNA booster dose 120–179 days before symptom onset, VE was 43% (95% CI: −11 to 71), 34% (95% CI: 13–50) and 27% (95% CI: −28 to 58), respectively. Our results showed no protection ≥ 180 days post booster for all products combined (Table 2).

### Analysis 4: Vaccine effectiveness by age group, overall and by time since mRNA booster dose 

We estimated VE among those who had received a first booster dose for the intervals 14–59, 60–119, 120–179 and ≥ 180 days from first booster dose to symptom onset. For all PSV products combined, VE after receipt of first mRNA booster dose 14–59 days before onset was at least 81% in each age group (20–59, 60–79 and ≥ 80 years). In the two older age groups (≥ 60 years), the VE for first mRNA booster received 60–119 days before symptom onset was at least 70%, while in the youngest age group (20–59 years) mRNA booster dose VE was 59%. For those vaccinated 120–179 days before onset, the VE point estimates for all age groups were ≤ 40%, with no protection observed for any age group when the booster was received ≥ 180 days before onset (Table 2).

### Supplementary analyses

We estimated the VE of mRNA booster dose by Omicron sub-lineage (BA.1, BA.2, BA.4/5) dominance periods, estimated using country-specific week of swab as proxy. The VE for mRNA booster dose (all PSV products combined) for Omicron sub-lineage BA.1 was > 75% overall and across all age groups and was highest in those aged ≥ 80 years. For BA.2 and BA.4/5, the VE was 25% and 13% overall, respectively. Sample size was very low for BA.4/5, resulting in very wide confidence intervals and precluding some stratified analyses. The VE by time since vaccination for these sub-lineages were greater in those whose last vaccination was < 60 days before onset than in those with vaccination ≥ 60 days, and the VE at < 60 days was lower for the later sub-lineages (87% and 41% for BA.1 and BA.2, respectively; BA.4/5 sample size was too small to complete this analysis as most vaccinated patients were vaccinated ≥120 days before onset). For the details of these results by sublineage, we refer to Supplementary Tables S2 and S3.

### Sensitivity analyses

Excluding 463 cases and 281 controls with known prior (PCR-confirmed) infection in those receiving complete PSV alone produced slightly greater VE for all products combined: from 1 (PSV only) to 5 percentage points higher for those with booster dose vaccination. The VE point estimates by age group for those who had only received complete PSV ranged from 13 percentage points lower (for those 20–59 years old) to 2 percentage points higher (in those aged 60–79 years) and 12 percentage points higher (in those aged ≥ 80 years). Booster dose VE in those without known prior infection ranged from 1 percentage point higher in the youngest age group to 7 percentage points higher in those aged 60–79 years. The data for these sensitivity analyses are accessible in Supplementary Table S4 (Analysis 1).

In the group who were either admitted to ICU or died in hospital (severe outcomes), and were in the BDTG, the VE for complete PSV in those ≥ 150 days from their last PSV dose was 14 percentage points higher than in the main analysis for this group. For those aged ≥ 80 years, although VE was 43 percentage points higher than in the main analysis, numbers were small and 95% CIs wide (sample size was too small to estimate VE for the younger age groups). For those receiving a booster dose ≥ 150 days from their last PSV dose, the VE was 3–5 percentage points different from the main analysis, overall and for each age group. For the detailed analyses we refer to Supplementary Table S4 (Analysis 2).

## Discussion

Pooled results from this multi-country European study of VE against hospitalisation with COVID-19 showed that the overall VE was ≤ 50% for all PSV products among hospitalised SARI patients aged ≥ 20 years who were eligible for but not receiving an mRNA booster dose during the Omicron circulating period (December 2021–July 2022) and who received their last PSV dose ≥ 150 days before symptom onset. In those who received an mRNA booster dose, VE increased to 54% (95% CI: 45–62), 60% (95% CI: 41–72) and 69% (95% CI: 54–79), respectively, for Comirnaty, Spikevax and Vaxzevria as PSV. Analysis by time since vaccination with mRNA booster (three 60-day periods from last booster dose to onset) for all PSV products combined indicated that the VE for the first mRNA booster dose decreased in each 60-day period, from 85% and 70% in the first two periods, to a much reduced 36% in the period 120–179 days between mRNA booster dose and symptom onset. The VE in those with onset 14–59 days after their first mRNA booster was ≥ 75% regardless of individual PSV product but was < 30% for those with onset ≥ 180 days after their first mRNA booster dose. We observed only small differences in VE point estimates (with overlapping 95% CIs) for those who received mRNA PSV and booster products vs those with non-mRNA PSV product followed by mRNA booster. Our study showed that for those SARI patients who were eligible for a booster but had not yet received this, several months (median > 200 days for both cases and controls) had passed between their last PSV dose and symptom onset. The lower VE in these individuals is likely to reflect waning immunity in the absence of a booster dose.

Our results should be interpreted with the following strengths and limitations in mind. Firstly, as with any multi-country study using pooled data, heterogeneity across countries is expected, from vaccine roll-out timing, coverage and product use, and SARS-CoV-2 variant distribution, to healthcare-seeking behaviour and case management strategies. To counter some of these, an adapted core protocol was in use, with homogeneous methodology applied across participating countries. Although residual and unmeasured confounding may be present and hence unaccounted for, most sites collected data prospectively, documenting important information on key confounders. For those receiving complete PSV alone, we estimated VE for those with onset < 150 days and ≥ 150 days from last PSV dose, to allow better comparability with those receiving a booster, as a booster dose was recommended in participating countries on average ca 150 days from last PSV dose. Product-specific VE estimates showed overlapping confidence intervals and the results did not demonstrate significant differences in protection against hospitalisation with PCR-confirmed SARS-CoV-2.

Combining individual-level data from several countries permitted a larger sample size to provide robust results for continued monitoring of VE against severe disease across Europe. However, sample sizes were low by individual vaccine product, limiting the precision of some estimates and preventing subgroup analyses among those aged 20–59 years and those with no chronic conditions.

We did not account for previous infection in either cases or controls (although sensitivity analyses showed 1–13 percentage point higher VE when those with known prior infection were excluded). However, as not all sites collect this information, and as some patients will have had mild or even asymptomatic prior infection not captured by our study, it is difficult to fully interpret the results. Measuring VE without knowledge of prior infection is a major challenge. In some participating countries, individuals with a SARS-CoV-2 infection have their subsequent COVID-19 vaccination dose delayed. Self-testing for SARS-CoV-2 has become common in many countries; it is difficult for hospital staff to document the number of prior infections, with dates, for each included patient (some of whom may have had more than one prior infection during 2022 alone). Of the 463 cases and 281 controls in our study with known, PCR-confirmed prior infection, the proportion unvaccinated was similar to those with no known, PCR-confirmed prior infection for cases (32% vs 31%, respectively) but was lower for controls (20% vs 14%, respectively). Further sensitivity analyses adjusting by known prior infection are planned for the future, on larger datasets with fewer missing this information. As sequencing results were not available for most SARI patients, we used proxy dates for when ≥ 80% samples sequenced by a country were Omicron; a process which has been used in other studies [33,34].

Other studies have also shown a positive effect of booster doses in preventing COVID-19 hospitalisation, although our results were lower than most (e.g. we found an all-product VE of 70% at 60–119 days, 27% ≥ 120 days from booster dose, vs 89% within 60 days and 66% at ≥ 120 days [35]). Another multi-country study during the Omicron period with a similar test-negative design pooled data from several Latin American countries. They showed Comirnaty VE of 25% without booster among those ≥ 65 years [36]. Our Comirnaty VE for those ≥ 65 years is potentially higher, as we show VE of 53% and 27% for those 60–79 and ≥ 80 years, respectively. With booster, the Latin American study had more comparable Comirnaty VE results to ours, however, at 56% among those ≥ 65 years vs 52% in 60–79 and 54% in ≥ 80-year-olds in our study [36]. There are several reasons which could explain our low observed VE. One could be our use of a sensitive SARI case definition [28], potentially leading to more cases with milder disease being recruited. This could result in inclusion of hospitalised patients ‘with’ COVID-19 rather than only those hospitalised ‘due to’ COVID-19. This also explains the lower VE found overall in our study compared with others, as our VE estimates were more similar to those found against infection than against hospitalisation [37]. Our sensitivity analysis using more severe hospitalised outcomes (ICU or death in hospital) was difficult to interpret due to low sample size, possibly due to the milder effect of the Omicron variant, which is known to have caused fewer hospitalisations [38,39]. Booster-vaccinated patients in our study had all received an mRNA booster dose (Comirnaty or Spikevax), and most patients with PSV had also received an mRNA vaccination; our results therefore reflect mainly receipt of homologous mRNA PSV and booster vaccination. Despite the lower level of effectiveness, however, our study (like others) shows that the booster dose improves VE and hence has the potential to decrease hospital burden and improve outcomes [35,40-42].

Evolving evidence based on early VE data and analysis of antibody levels after the first booster dose suggest that there is gradual waning of immunity against the Omicron variant. Single-country studies in the United States (US) [35,43-45], Denmark [46] and Canada [47] undertaken during the early phase of Omicron emergence with short follow-up periods reported high VE of the booster dose (> 80%) against severe outcomes of Omicron infection. Other studies (some with the same test-negative design as ours) confirmed these early findings. For example, VE against COVID-19 hospitalisation and death was in the range of 70–80% any time after the second PSV dose and > 90% after the first booster dose in Qatar [48]. In the US, Tartof et al. reported VE after three doses of Comirnaty of 85% (95% CI: 80–89) against hospital admission due to the Omicron variant within 90 days, falling to 55% (95% CI: 28–71) ≥ 90 days [15]. Other study results indicated rapid waning of this effect with increasing time from vaccination [37,46,47,49-51], starting from 90 days after booster administration. Our results showing decreasing VE over time during the Omicron period, with no protection observed from 6 months after the booster dose, underscore the importance of monitoring VE against hospitalisation with this variant. This has policy implications for European countries in terms of recommendations for timing of booster doses.

This study provides some evidence of waning effectiveness, but the lower VE observed in patients with onset from 120 days and particularly from 180 days after first mRNA booster dose than in those with first mRNA booster dose < 60 days from onset could also be due to the different sub-lineages circulating at the time of their illness. In supplementary analyses, VE was significantly higher in those receiving an initial booster dose for the much earlier BA.1 period (from the study start in December 2021 to February 2022) than the BA.2 (from March to May 2022) or BA.4/5 (from June to study end in July 2022) variants. Our study also showed that VE was lower with greater time since last booster dose within each sub-lineage period.

Measuring only overall VE against Omicron may mask differential VE in different sub-lineage periods and over time, with the low overall VE due in part to many patients having long lags since their last booster dose (particularly for older individuals, who would have been vaccinated earlier) and the emergence of sub-lineages with greater ability for immune escape. The emergence of Omicron sub-lineages BA.5 and, to a lesser extent, BA.4, did raise concern about high transmissibility [52] and partial escape of natural and vaccine-induced immunity [53-55]. These variants have rapidly become dominant in the EU/EEA. For countries participating in our study, BA.4/5 became dominant only towards the end of our study period, from 10 June 2022. A higher sample size for BA.2 and BA.4/5 is needed to better disentangle the effects of time since vaccination and VE against Omicron sub-lineages.

Countries have progressively introduced additional booster doses in vulnerable groups, and EU/EEA countries recommended a second booster in immunocompetent individuals aged 60 years and older in late 2022 [21], when vaccination campaigns focused on administering adapted bivalent booster vaccines. Although subsequent boosters may partly restore the immunity and increase protection conferred by the earlier doses of the vaccine [56,57], the speed of waning immunity needs further study, along with attempts to disentangle the competing effects of age, variant sub-lineage, prior infection and time since vaccination.

## Conclusion

Our results suggest that, during dominant circulation of the Omicron variant in participating countries, among adults aged ≥ 20 years, overall VE for complete PSV without booster, ≥ 150 days after last PSV dose, was ≤ 50% for the three main vaccine products in use. Higher VE after administration of a booster dose suggests beneficial effect of booster vaccination during this period, particularly for those with onset within 120 days of their booster dose. Although the policy implications of these findings are potentially substantial, there is a need for confirmatory findings from research groups in other countries.

## Supplementary Data

286000 Supplement
