# Supplementary material for: Vaccine effectiveness against COVID-19 hospitalisation in adults (≥ 20 years) during Omicron-dominant circulation: I-MOVE-COVID-19 and VEBIS SARI VE networks, Europe, 2021 to 2022
Source: Euro Surveill. 2023 Nov 23;28(47):2300187. doi: 10.2807/1560-7917.ES.2023.28.47.2300187 (PMC10668256; doi:10.2807/1560-7917.ES.2023.28.47.2300187)
Supplement: Supplement [file 23-00187_ROSE_Supplement.pdf]

This supplementary material is hosted by *Eurosurveillance* as supporting information alongside the article "Vaccine effectiveness against COVID-19 hospitalisation in adults (≥ 20 years) during Omicron-dominant circulation: I-MOVE-COVID-19 and VEBIS SARI VE networks, Europe, 2021", on behalf of the authors, who remain responsible for the accuracy and appropriateness of the content. The same standards for ethics, copyright, attributions and permissions as for the article apply. Supplements are not edited by *Eurosurveillance* and the journal is not responsible for the maintenance of any links or email addresses provided therein.

Supplementary Table S1. Effectiveness of COVID-19 complete PSV against hospitalisation among adults (≥20 years) by age group and time since last PSV dose for all vaccine products, I-MOVE-COVID-19 and VEBIS hospital networks, Europe, 13 Dec 2021–31 Jul 2022 (n=5115[a])

**Main analysis 1: Vaccine effectiveness <150 days from receipt of last PSV dose to symptom onset in those not eligible for booster dose, by age group**			
**PSV vaccine product**		**Vaccinated/unvaccinated cases; Vaccinated/unvaccinated controls**	**VE[b] (95% CI)**
**Any PSV vaccine product (eight sites;[c] N=1245[c])**			
All 14–149 days		67/791; 48/339	51 (21–69)
Age group			
	20–59	27/163; 19/88	46 (-19–75)
	60–79	21/334; 22/153	72 (37–88)
	≥80	19/294; 7/98	15 (-122–69)
**Main analysis 1: Vaccine effectiveness <150 days from receipt of last PSV dose to symptom onset in those not eligible for booster dose, by time since last PSV dose (14–59, 60–119, 120–149 days from receipt of dose to symptom onset)**			
**Number of days from last PSV to onset**		**Vaccinated/unvaccinated cases; Vaccinated/unvaccinated controls**	**VE[b] (95% CI)**
**Any PSV vaccine product (eight sites;[d] N=1245[a])**			
All 14–149 days		67/791; 48/339	51 (21-68))
Days from last PSV dose to onset			
	14–59	23/791; 13/339	54 (-4–80)
	60–119	27/791; 20/339	55 (11–77)
	120–149	17/791; 15/339	40 (-35–73)

I-MOVE: Influenza – Monitoring vaccine effectiveness in Europe; PSV: primary series vaccination; VE: vaccine effectiveness; VEBIS: Vaccine Effectiveness, Burden and Impact Studies.

[a]N=5115 after dropping 26 with a second booster (sample size too small to analyse second booster).

[b]Odds ratios adjusted by country, time (restricted cubic spline of swab date or swab month as categorical variable, depending on model), age (restricted cubic spline or age as linear variable, depending on model), sex, presence/absence of chronic condition (asthma, diabetes, heart disease, lung disease).

[c]N=1245 after dropping 2987 records of patients who received a first mRNA booster dose, 26 with a second booster, 776 with last primary series vaccination dose ≥150 days from onset and 107 from three sites with fewer than five cases or controls, or <20 cases and controls.

[d]Eight sites: Belgium, Croatia, France, Ireland, Malta, the Netherlands, Portugal and Spain.

Supplementary Table S2. Effectiveness of COVID-19 complete PSV and mRNA booster dose vaccination against hospitalisation among adults (≥20 years) by age group for Omicron sub-lineages BA.1, BA.2 and BA.4/5, I-MOVE-COVID-19 and VEBIS hospital networks, Europe, 13 Dec 2021–31 Jul 2022[a] (n=3,485[b])

**Supplementary analysis 1: Vaccine effectiveness in those eligible for first booster dose by age group. Vaccination ≥ 150 days before onset for patients with PSV only and ≥ 150 days between last PSV and mRNA booster dose (for those with PSV and mRNA booster), Omicron sub-lineage BA.1**

PSV product	Cases vaccinated/unvaccinated; Controls vaccinated /unvaccinated		VE[c] (95% CI)	
	Complete PSV only	Complete PSV plus booster	Complete PSV only	Complete PSV plus booster
**Any PSV product**	**Four sites;[d] N=813**	**Eight sites;[e] N=990**		
All ≥20	239/376; 140/58	265/423; 237/65	70 (54–80)	79 (68–86)
Age group				
20–59	59/70; 44/16	21/86; 23/19	76 (43–90)	78 (39–92)
60–79	114/182; 64/26	94/205; 93/29	64 (32–80)	79 (60–89)
≥80	66/124; 32/16	150/132; 121/17	64 (24–83)	80 (58–91)
Chronic condition[f]				
No	79/168; 44/23	62/196; 35/25	75 (50–88)	70 (34–86)
Yes	160/208; 96/35	203/227; 202/40	67 (46–80)	82 (70–89)

Supplementary analysis 2: Vaccine effectiveness in those eligible for first booster dose by age group. Vaccination ≥ 150 days before onset for patients with PSV only and ≥ 150 days between last PSV and mRNA booster dose (for those with PSV and mRNA booster), Omicron sub-lineage BA.2

PSV product	Cases vaccinated/unvaccinated; Controls vaccinated /unvaccinated		VE[c] (95% CI)	
	Complete PSV only	Complete PSV plus booster	Complete PSV only	Complete PSV plus booster
**Any PSV product**	**Three sites;[g] N=331**	**Six sites;[h] N=1,006**		
All ≥20	52/90; 75/114	313/102; 468/123	-4 (-68–36)	25 (-8–48)
Age group				
20–59	12/21; 36/25	21/25; 55/30	61 (0–85)	51 (-16–79)
60–79	19/32; 23/58	117/38; 206/59	-84 (-315–18)	25 (-34–58)
≥80	21/37; 16/31	175/39; 207/34	-19 (-192–52)	18 (-53–56)
Chronic condition[f]				
No	15/31; 30/40	70/38; 88/42	39 (-41–74)	50 (-4–76)
Yes	37/59; 45/74	243/64; 380/81	-32 (-145–29)	18 (-26–47)

Supplementary analysis 3: Vaccine effectiveness in those eligible for first booster dose by age group. Vaccination ≥ 150 days before onset for patients with PSV only and ≥ 150 days between last PSV and mRNA booster dose (for those with PSV and mRNA booster), Omicron sub-lineage BA.4/5

Any PSV product	Cases vaccinated/unvaccinated; Controls vaccinated /unvaccinated		VE[c] (95% CI)	
	Complete PSV only	Complete PSV plus booster	Complete PSV only	Complete PSV plus booster
**Any PSV product**	**Two sites;[i] N=155**	**Three sites;[j] N=466**		
All ≥20	26/80; 17/32	233/81; 117/32	26 (-65–67)	13 (-7–48)
Age group				
20–59	1/10; 7/9	10/10; 11/9	NC[k]	NC[k]
60–79	12/22; 6/10	76/23; 38/10	NC	18 (-118–69)
≥80	13/48; 4/13	150/48; 68/13	NC	25 (-66–66)
Chronic condition[f]				
No	6/25; 5/7	49/26; 11/7	NC	40 (-147–85)
Yes	20/55; 12/25	187/55; 106/25	6 (-144–63)	4 (-76–47)

I-MOVE: Influenza – Monitoring vaccine effectiveness in Europe; NC: Not calculated; PSV: primary series vaccination; VE: vaccine effectiveness; VEBIS: Vaccine Effectiveness, Burden and Impact Studies.

[a]From the first date of Omicron BA.1 dominance (defined as first day of the first week when ≥80% BA.1 was identified from samples sequenced in any participating country) to the last date of the study.

[b]N=3485 after dropping all those not in either Omicron BA.1, BA.2 or BA.4/5 dominant periods.

[c]Odds ratios adjusted by country, time (restricted cubic spline of swab date or swab month as categorical variable, depending on model), age (restricted cubic spline or age as linear variable, depending on model), sex, presence/absence of chronic condition (asthma, diabetes, heart disease, lung disease).

[d]Four sites: Croatia, the Netherlands, Portugal and Spain.

[e]Eight sites: Belgium, Croatia, France, Ireland, Navarra, the Netherlands, Portugal and Spain.

[f]In analyses stratified by chronic condition, the adjustment for presence/absence of chronic condition was removed.

[g]Three sites: Belgium, Croatia and Spain.

[h]Six sites: Belgium, Croatia, France, Ireland, Navarra and Spain.

[i]Two sites: Croatia and Spain.

[j]Three sites: Croatia, Navarra and Spain.

[k]Numbers are too small to provide robust VE estimates (penalised logistic regression showed evidence of small sample bias).

Supplementary Table S3. Effectiveness of COVID-19 booster vaccination against hospitalisation among adults (≥20 years) for all vaccine products combined by time since vaccination, for Omicron sub-lineages BA.1, BA.2, and BA.4/5, I-MOVE-COVID-19 and VEBIS hospital networks, Europe, 27 Dec 2021–31 Jul 2022[a] (n=2,383[b])

**Supplementary analysis 4: Vaccine effectiveness by time (number of days) since receipt of booster dose (14–60, 60–119, ≥ 120 days from receipt of first mRNA booster dose to symptom onset), Omicron sub-lineage BA.1**			
		**Vaccinated/unvaccinated cases; Vaccinated/unvaccinated controls**	**VE[c] (95% CI)**
**Any PSV product (eight sites;[d] N=918)**			
All		254/407; 205/52	78 (65–86)
Days from first booster dose to onset (among vaccinated cases and controls)	*14–59*	78/407; 96/52	87 (78–93)
	60–119	156/407; 105/52	73 (54–84)
	≥120	20/407; 4/52	-65 (-484–53)
**Supplementary analysis 5: Vaccine effectiveness by time (number of days) since receipt of booster dose (14–60, 60–119, ≥ 120 days from receipt of first mRNA booster dose to symptom onset), Omicron sub-lineage BA.2**			
		**Vaccinated/unvaccinated cases; Vaccinated/unvaccinated controls**	**VE[c] (95% CI)**
**Any PSV product (six sites;[e] N=1,006)**			
All		313/102; 468/123	25 (-7–48)
Days from first booster dose to onset (among vaccinated cases and controls)	*14–59*	8/102; 17/123	41 (-63–79)
	60–119	50/102; 133/123	50 (18–70)
	≥120	255/102; 318/123	19 (-21–45)
**Supplementary analysis 6: Vaccine effectiveness by time (number of days) since receipt of booster dose (14–60, 60–119, ≥ 120 days from receipt of first mRNA booster dose to symptom onset), Omicron sub-lineage BA.4/5**			
		**Vaccinated/unvaccinated cases; Vaccinated/unvaccinated controls**	**VE[c] (95% CI)**
**Any PSV product (three sites;[f] N=459)**			
All		233/81; 113/32	13 (-50–50)
Days from first booster dose to onset (among vaccinated cases and controls)	*14–59*	2/81; 1/32	NC
	60–119	0/81; 3/32	NC
	≥120	231/81; 109/32	11 (-53–49)

I-MOVE: Influenza – Monitoring vaccine effectiveness in Europe; NC: Not calculated; PSV: primary series vaccination; VE: vaccine effectiveness; VEBIS: Vaccine Effectiveness, Burden and Impact Studies.

[a]From the first date of Omicron BA.1 dominance (defined as first day of the first week when ≥80% BA.1 was identified from samples sequenced in any participating country) to the last date of the study.

[b]N=2,383 after dropping all those not in either Omicron BA.1, BA.2 or BA.4/5 dominant periods.

[c]Odds ratios adjusted by country, time (restricted cubic spline of swab date or swab month as categorical variable, depending on model), age (restricted cubic spline or age as linear variable, depending on model), sex, presence/absence of chronic condition (asthma, diabetes, heart disease, lung disease).

[d]Eight sites: Belgium, Croatia, France, Ireland, Navarra, the Netherlands, Portugal and Spain.

[e]Six sites: Belgium, Croatia, France, Ireland, Navarra and Spain.

[f]Three sites: Croatia, Navarra and Spain.

Supplementary Table S4. Sensitivity analyses: Effectiveness of COVID-19 complete PSV with and without mRNA booster dose against hospitalisation among adults (≥20 years) in patients with (a) no known prior infection and (b) severe outcomes only, all vaccine products combined, I-MOVE-COVID-19 and VEBIS hospital networks, Europe, 13 Dec 2021–31 Jul 2022

Sensitivity analysis 1: Vaccine effectiveness of PSV ≥150 days from receipt of last PSV dose and PSV plus mRNA booster dose, with booster received ≥150 days from receipt of last PSV dose (all products combined), overall and by age group; SARI patients with no known prior infection only

	Vaccinated/unvaccinated cases; Vaccinated/unvaccinated controls		VE[a] (95% CI)	
	Complete PSV only	Complete PSV plus mRNA booster	Complete PSV only	Complete PSV plus mRNA booster
**Any PSV product**	**11 sites;[b] N=1702[c]**	**10 sites;[d] N=3381[c]**		
All ≥20	363/737; 297/305	1057/671; 1352/301	44 (29–55)	64 (57–71)
Age group				
20–59	92/145; 113/87	73/131; 153/84	52 (26–69)	67 (48–79)
60–79	162/315; 123/132	391/283; 596/131	40 (14–58)	65 (53–74)
≥80	109/277; 61/86	593/257; 603/86	41 (8–62)	64 (50–74)

Sensitivity analysis 2: Vaccine effectiveness of PSV ≥150 days from receipt of last PSV dose and PSV plus mRNA booster dose, with booster received ≥150 days from receipt of last PSV dose (all products combined), overall and by age group; SARI patients with severe outcomes only (admitted to intensive care or in-hospital death)

	Vaccinated/unvaccinated cases; Vaccinated/unvaccinated controls		VE[a] (95% CI)	
	Complete PSV only	Complete PSV plus mRNA booster	Complete PSV only	Complete PSV plus mRNA booster
**Any PSV product**	**11 sites;[b] N=403[e]**	**10 sites;[d] N=592[e]**		
All ≥20	88/247; 33/35	182/240; 135/35	57 (19–77)	54 (17–74)
Age group				
20–59	13/30; 8/5	11/29; 11/5	NC[f]	NC[f]
60–79	39/107; 13/17	67/103; 55/17	NC[f]	53 (-17–81)
≥80	36/110; 12/13	104/108; 69/13	72 (21–90)	55 (-14–82)

I-MOVE: Influenza – Monitoring vaccine effectiveness in Europe; NC: Not calculated; PSV: primary series vaccination; SARI: severe acute respiratory infection; VE: vaccine effectiveness; VEBIS: Vaccine Effectiveness, Burden and Impact Studies.

[a]Odds ratios adjusted by country, time (restricted cubic spline of swab date or swab month as categorical variable, depending on model), age (restricted cubic spline or age as linear variable, depending on model), sex, presence/absence of chronic condition (asthma, diabetes, heart disease, lung disease).

[b]Eleven sites: Belgium, Croatia, France, Ireland, Lithuania, Malta, Navarra, the Netherlands, Portugal, Romania and Spain.

[c]N=1702 and 3381 after dropping all records of patients with known prior infection in those with primary series vaccination (PSV) only, and in those with PSV plus mRNA booster dose, respectively.

[d]Ten sites: Belgium, Croatia, France, Ireland, Lithuania, Malta, Navarra, the Netherlands, Portugal and Spain.

[e]N=403 and 592 after dropping all records of patients who did not have a severe outcome (or with this information missing), in those with PSV only, and in those with PSV plus mRNA booster dose, respectively.

[f]Numbers are too small to provide robust VE estimates (penalised logistic regression showed evidence of small sample bias).
